# Supplementary material for: Perimenopausal state oestradiol to progesterone imbalance drives Alzheimer’s risk via ERRα dysregulation and energy dyshomeostasis
Source: Nat Commun. 2025 Nov 22;16:11546. doi: 10.1038/s41467-025-66726-4 (PMC12748969; doi:10.1038/s41467-025-66726-4)
Supplement: Supplementary file 1 — Supplementary Information [file 41467_2025_66726_MOESM1_ESM.pdf]

Supplementary Information

Perimenopausal State Oestradiol to Progesterone Imbalance Drives Alzheimer's Risk via ERRα Dysregulation and Energy Dyshomeostasis

Jacquelyne Ka-Li SUN[1], Amy Zexuan PENG[1], Ronald P. HART[2], Karl HERRUP[3], Deng WU[1], Genper Chi-Ngai WONG[1], Kim Hei-Man CHOW[1,4#]

[1] School of Life Sciences, Faculty of Science, The Chinese University of Hong Kong, Hong Kong

[2] Department of Cell Biology and Neuroscience, Rutgers University, Piscataway, New Jersey, USA

[3] Department of Neurobiology, School of Medicine, University of Pittsburgh, Pittsburgh, Pennsylvania, USA.

[4] Gerald Choa Neuroscience Institute, The Chinese University of Hong Kong, Hong Kong

# To whom correspondence should be addressed:
Kim Hei-Man CHOW
Email: heimanchow@cuhk.edu.hk
Phone: (+852)3943-1530

Table of Contents

Supplementary Fig. 1. Female patients with late-onset Alzheimer's disease (LOAD) exhibit more pronounced changes in their transcriptomic profiles.

Supplementary Fig. 2. *ESRRA* and *PPARG1A* gene and protein expression levels in LOAD versus ND samples of different sexes.

Supplementary Fig. 3. Additional data related to behavioural tests and their correlations with plasma hormonal changes.

Supplementary Fig. 4. ERR and PGC1α protein expression levels in VCD-treated C57BL/6 mice.

Supplementary Fig. 5. Additional data related to the computational simulation and docking of the ERRα ligand binding domain structure.

Supplementary Fig. 6. Unbiased metabolomics analysis of enriched neuronal and nonneuronal cell populations harvested from cerebral cortex tissues of vehicle- or VCD-treated (E2:P4 ≥ 2.5 pg/ng) C57BL/6 mice.

Supplementary Fig. 7. Additional data related to ChIP–PCR analysis.

Supplementary Fig. 8. Scatter plots generated from the Transcriptomics Explorer of the Allen Brain Map reveal the relative expression levels of target genes of interest in various mouse brain cell types within the whole cortex and hippocampus regions.

Supplementary Fig. 9. Tukey plots for comparisons of the expression levels of ERα-targeted metabolic genes in ROSMAP brain samples harvested from patients of different biological sexes with different cognitive scores (Cogdx).

Supplementary Fig. 10. Quantification of the Western blot data.

Supplementary Fig. 11. Additional data related to AAV-shRNA *in vivo* experiments.

Supplementary Fig. 12. Molecular profiling of enriched neuronal populations from cerebral cortex tissues harvested from control or *Esrra*-KD C57BL/6 mice.

Supplementary Fig. 13. Levels of key metabolites in brain tissues harvested from VCD-treated animals.

Supplementary Fig. 14. Permutation analysis confirmed that the differentially expressed metabolites found in female LOAD subjects in the ROSMAP cohort are not biased by the preexisting difference in sample numbers.

Supplementary Fig. 15. Analyses of the succinate dehydrogenase subunits *Sdha* and *Sdhd.*

Supplementary Fig. 16. Additional data related to behavioural tests and their correlations with plasma hormonal changes in 3xTg animals.

Supplementary Fig.17. Bulk transcriptomic analysis of brain tissues harvested from VCD-treated 3xTg mice at Cycles 14–15 and immunoblotting experiment quantification.

Supplementary Fig. 18. Changes in plasma and brain E2 and P4 levels upon P4 administration in C57BL/6 and 3xTg animals.

Supplementary Fig. 19. Additional data related to behavioural tests, immunohistochemistry analysis, and immunoblotting experiments in test animals subjected to 60 days of P4 supplementation at Cycles 14–15.

Supplementary Fig. 20. Bulk transcriptomic and metabolomic analyses of brain tissues harvested from VCD-treated 3xTg mice subjected to 60 days of P4 supplementation at cycles 14–15.

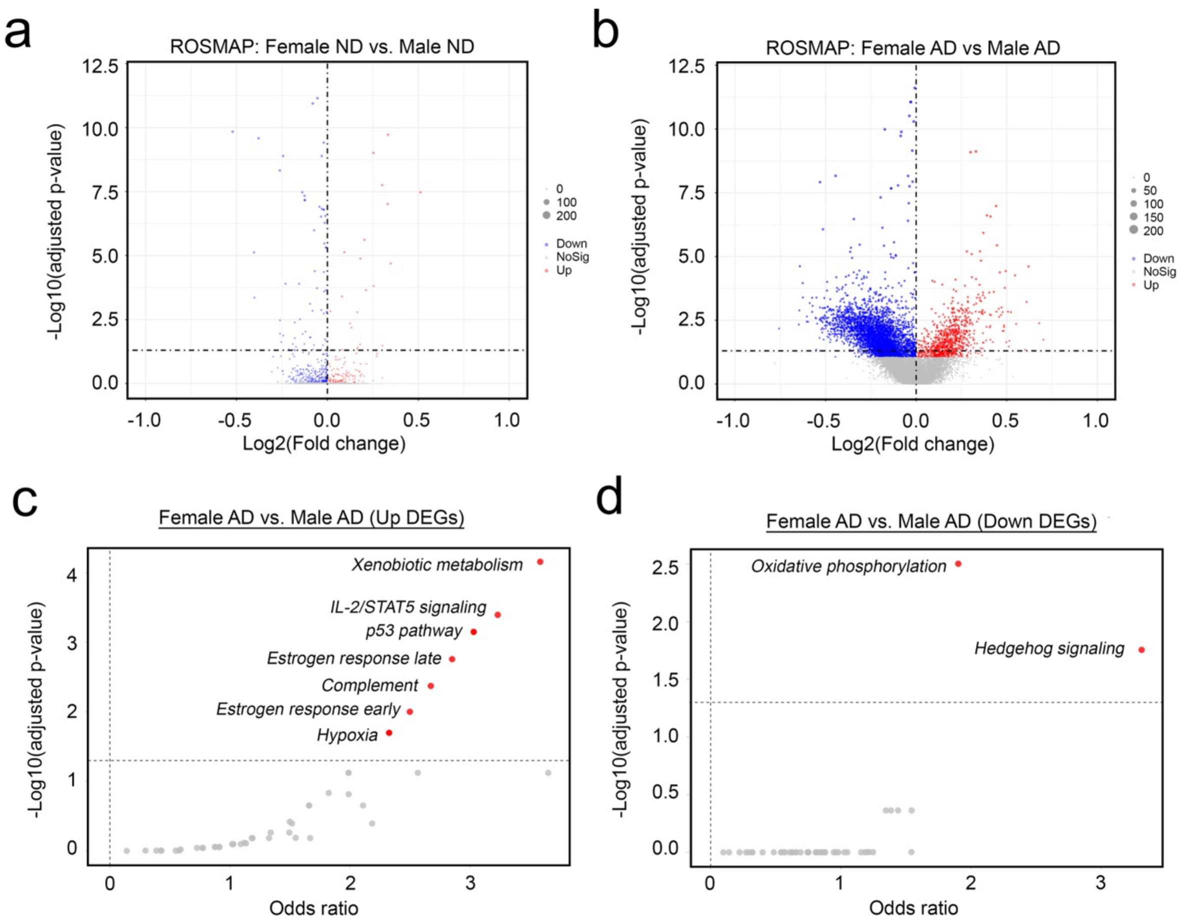

Supplementary Fig. 1. Female patients with late-onset Alzheimer's disease (LOAD) exhibit more pronounced changes in their transcriptomic profiles. a-b, Volcano plots illustrating DEGs curated from the comparison between **a.** ND or **b.** LOAD samples of different sexes (Limma with Benjamini–Hochberg correction). **c-d,** Functional enrichment analysis of **c.** upregulated or **d.** downregulated DEGs identified from $\mathrm{Female}_{\mathrm{LOAD}}$ versus $\mathrm{Male}_{\mathrm{LOAD}}$ samples performed on Enrichr[1] (two-tailed Fisher's exact test with correction). These figure panels supplement the analyses shown in Fig. 1b-c.

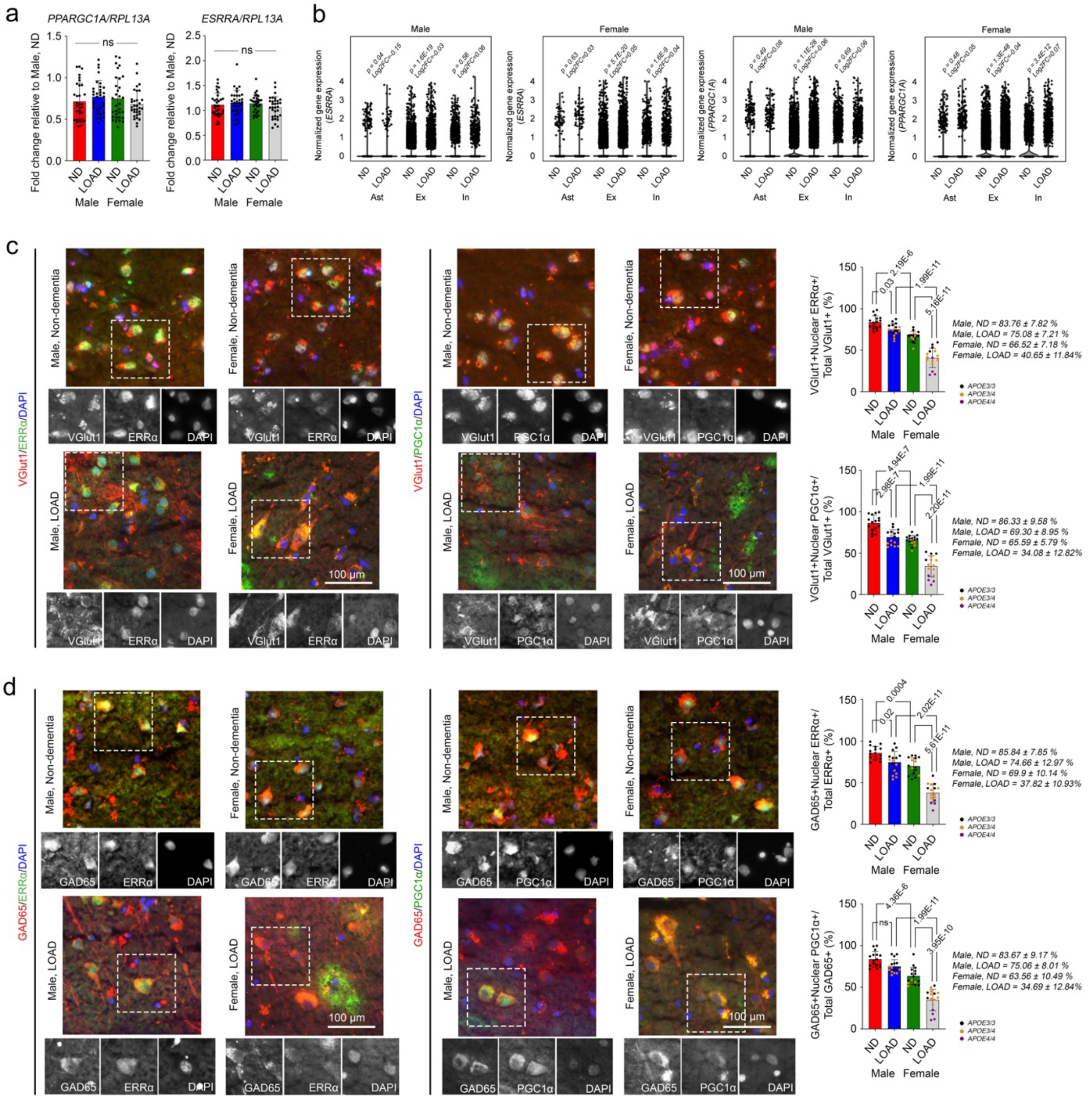

Supplementary Fig. 2. *ESRRA* and *PPARG1A* gene and protein expression levels in LOAD versus ND samples of different sexes. a, Quantitative PCR evaluation of the expression levels of *ESRRA* and *PPARG1A* in human patient prefrontal cortex samples (n=4; 8 technical replicates; Kruskal–Wallis test with Dunn's multiple comparisons test for *PPARG1A/RPL13A;* one-way ANOVA with Tukey's multiple comparisons test for *ESRRA/RPL13A*). **b,** Comparison of *ESRRA* and *PPARG1A* gene expression levels in various cell types in the prefrontal cortex (Brodmann area 10) single-nucleus transcriptomic data from a total of 48 LOAD (n=24) and ND (n=24), age (mean ± SD = 85.646 ± 4.215), and sex-matched (12 males and 12 females per group) patients[2] (Wilcoxon rank-sum test with Bonferroni correction). **c-d,** Representative immunofluorescence staining images of human prefrontal cortex tissues reveal changes in nuclear signals of ERRα or PGC1α in **c**. excitatory VGLUT1-positive neurons or **d.** GAD65-positive inhibitory neurons in LOAD versus ND samples of different sexes. Quantification is shown below (n=4; 4 technical repeats; one-way

ANOVA with Tukey's multiple comparisons test). N represents biological replicates. The values represent the mean ± s.d. Source data are provided as a Source Data file.

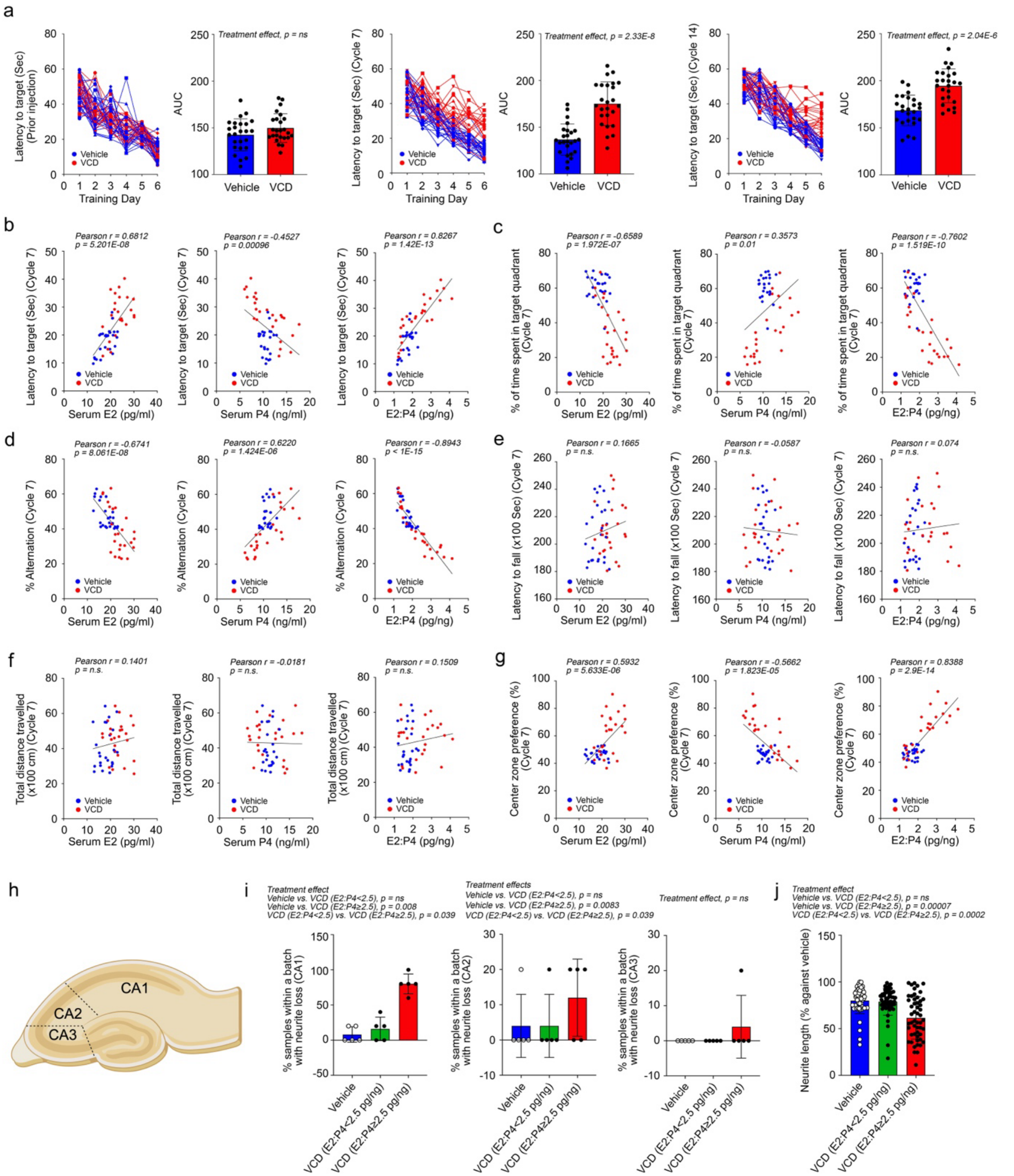

Supplementary Fig. 3. Additional data related to behavioural tests and their correlations with plasma hormonal changes. a, Latencies to target data during the 6-day training in the MWM paradigm conducted prior to treatment, Cycle 7 and Cycle 14. The areas under the curve were calculated and are presented on the right (n=25; two-tailed unpaired t test). **b-g,** Comparison of the strength of correlations between performance from various behavioural paradigms, including **b.** latency to target and **c.** percentage of time spent in the target quadrant of the MWM paradigm; **d.** percentage alternation of the Y-maze paradigm; **e.** latency to fall of the rotarod paradigm; **f.** total

distance travelled and **g.** centre zone preference of the open field test, with either the plasma E2, plasma P4 or E2:P4 ratio measured during the proestrus phase of Cycle 7 (n=25, Pearson correlation test). **h,** Schematic diagram showing the gross anatomy of the mouse hippocampus [Created in BioRender. Chow, K. (2025) https://BioRender.com/oml8opd]. **i,** Quantification of the percentage of samples within a cohort with neurite loss in different hippocampal regions (i.e., CA1, CA2 and CA3) was performed (n=5 cohorts with a total of 25 biological replicates; Kruskal–Wallis test with Dunn's multiple comparisons test). **j,** Quantification of neurite length in different treatment groups normalized against that in the vehicle control group in the CA1 region (n=10 biological replicates; 5–6 technical repeats; Kruskal–Wallis test with Dunn's multiple comparisons test). N represents biological replicates. The values represent the mean ± s.d. Source data are provided as a Source Data file.

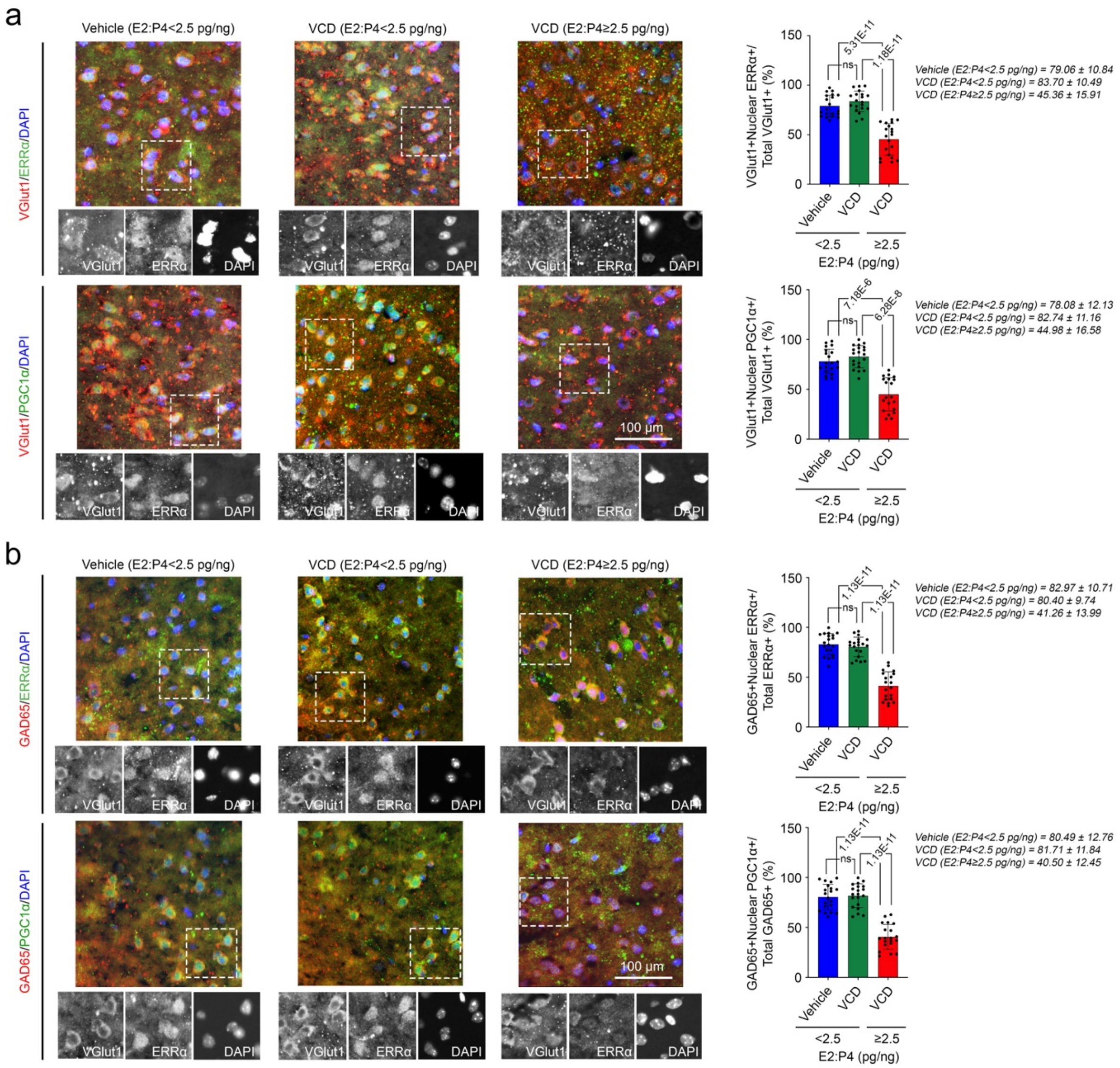

Supplementary Fig. 4. ERR and PGC1α protein expression levels in VCD-treated C57BL/6 mice. a-b, Representative immunofluorescence staining images of mouse prefrontal cortex tissues reveal changes in nuclear signals of **a,** ERRα or **b,** and PGC1α in excitatory VGLUT1-positive neurons or GAD65-positive inhibitory neurons. Quantification is shown below (n = 5 biological replicates, 4 technical replicates for each sample; one-way ANOVA with Tukey's multiple comparisons test for all except for the analysis of VGlut1+nucler PGC1α+ excitatory neurons, for which the Kruskal–Wallis test with Dunn's multiple comparison test was used). N represents biological replicates. The values represent the mean ± s.d. Source data are provided as a Source Data file.

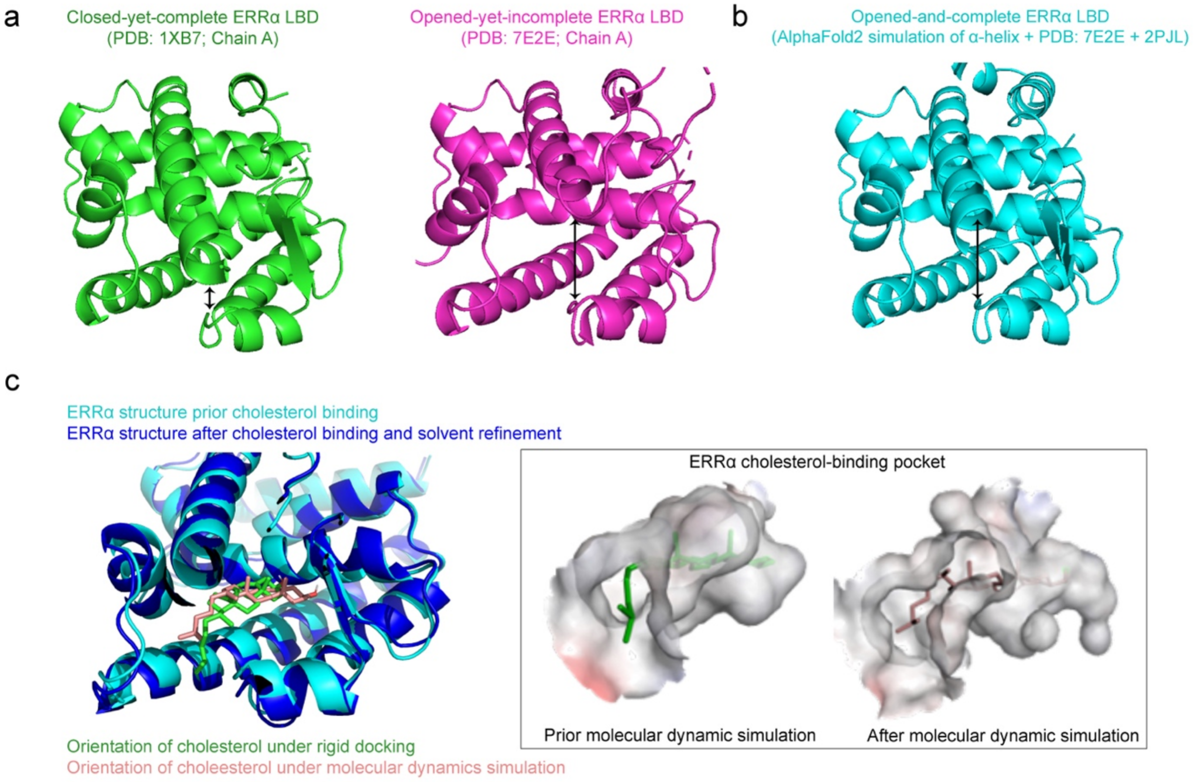

Supplementary Fig. 5. Additional data related to the computational simulation and docking of the ERRα ligand binding domain structure. a, Green: a closed-yet-complete ERRα ligand binding domain (LBD) extracted from the PDB: 1XB7, Chain A. Magenta: an opened-yet-incomplete ERRα LBD extracted from the PDB: 7E2E, Chain A. **b,** Open-and-complete ERRα LBD structure generated from combining the PDB: 7E2E, Chain A with the missing α-helix simulated by AlphaFold2. **c,** Comparison of the ERRα structure before or after cholesterol binding with solvent refinement. Dynamic simulation of the ERRα cholesterol binding pocket.

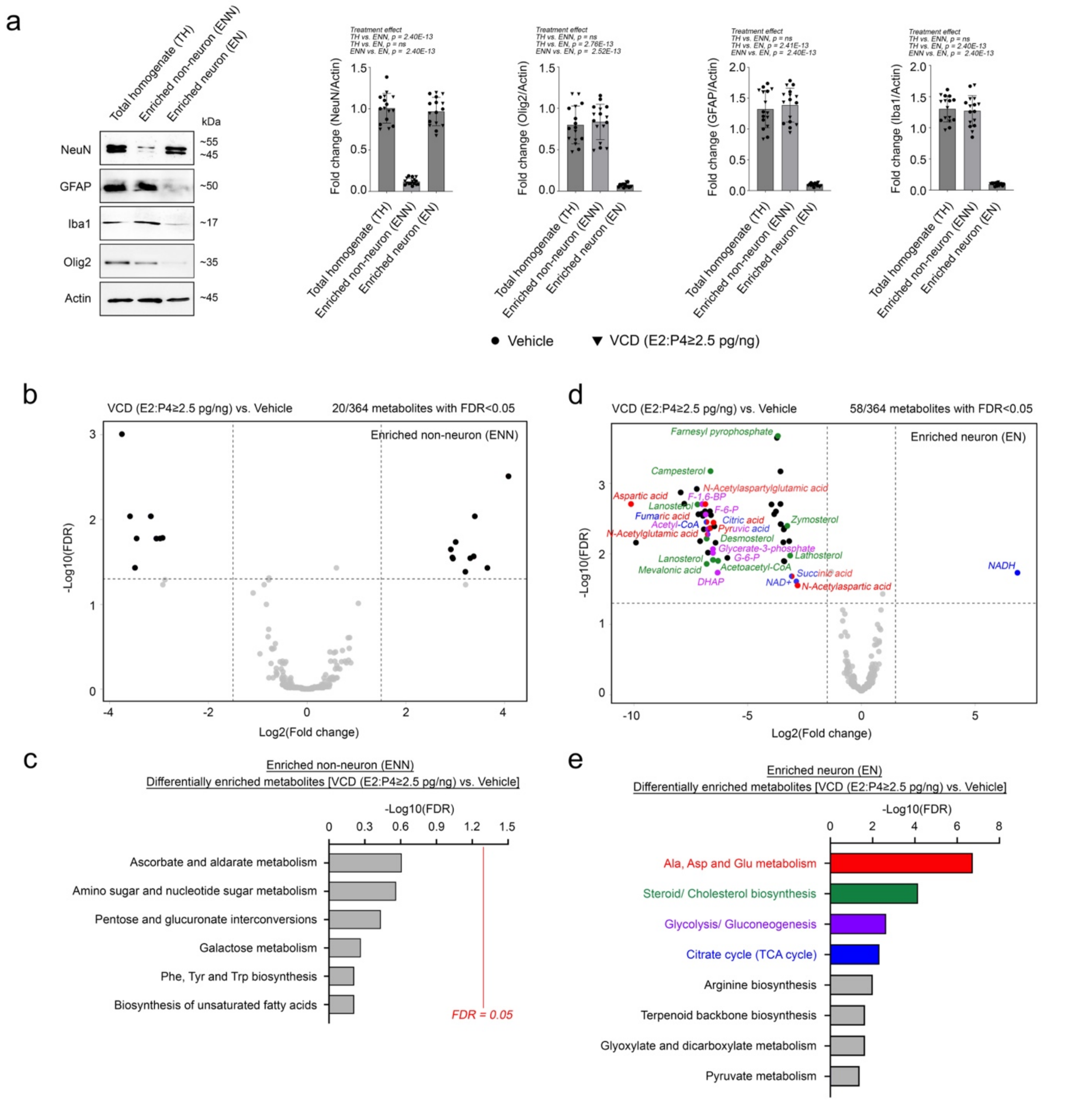

Supplementary Fig. 6. Unbiased metabolomics analysis of enriched neuronal and nonneuronal cell populations harvested from cerebral cortex tissues of vehicle- or VCD-treated (E2:P4 ≥ 2.5 pg/ng) C57BL/6 mice. a, Representative immunoblots showing successful enrichment of neuronal and nonneuronal glial cell populations from brain tissues of various samples (n=16; one-way ANOVA with Tukey's multiple comparison test). **b,** Volcano plot summarizing significantly altered metabolites in nonneuronal cells enriched from the brain tissues of VCD (E2:P4≥2.5 pg/ng) versus vehicle-treated animals (n=6; two-sided multiple unpaired t test with Benjamini, Krieger, and Yekutieli correction). **c,** Differentially changed metabolites were clustered and analysed by metabolite set enrichment analysis conducted on MetaboAnalyst[3]. Unique colour codes of the metabolites in **e.** are matched to the corresponding pathways (global test with Bonferroni correction[4]). **d,** Volcano plot summarizing significantly altered metabolites in

neuronal cells enriched from the brain tissues of VCD ($E2:P4 \geq 2.5$ pg/ng) versus vehicle-treated animals (n=6; two-sided multiple unpaired t test with Benjamini, Krieger, and Yekutieli correction). **e,** Differentially changed metabolites were clustered and analysed by metabolite set enrichment analysis on MetaboAnalyst[3]. Unique colour codes of the metabolites in **e.** are matched to the corresponding pathways (global test with Bonferroni correction[4]). N represents biological replicates. The values represent the mean ± s.d. Source data are provided as a Source Data file.

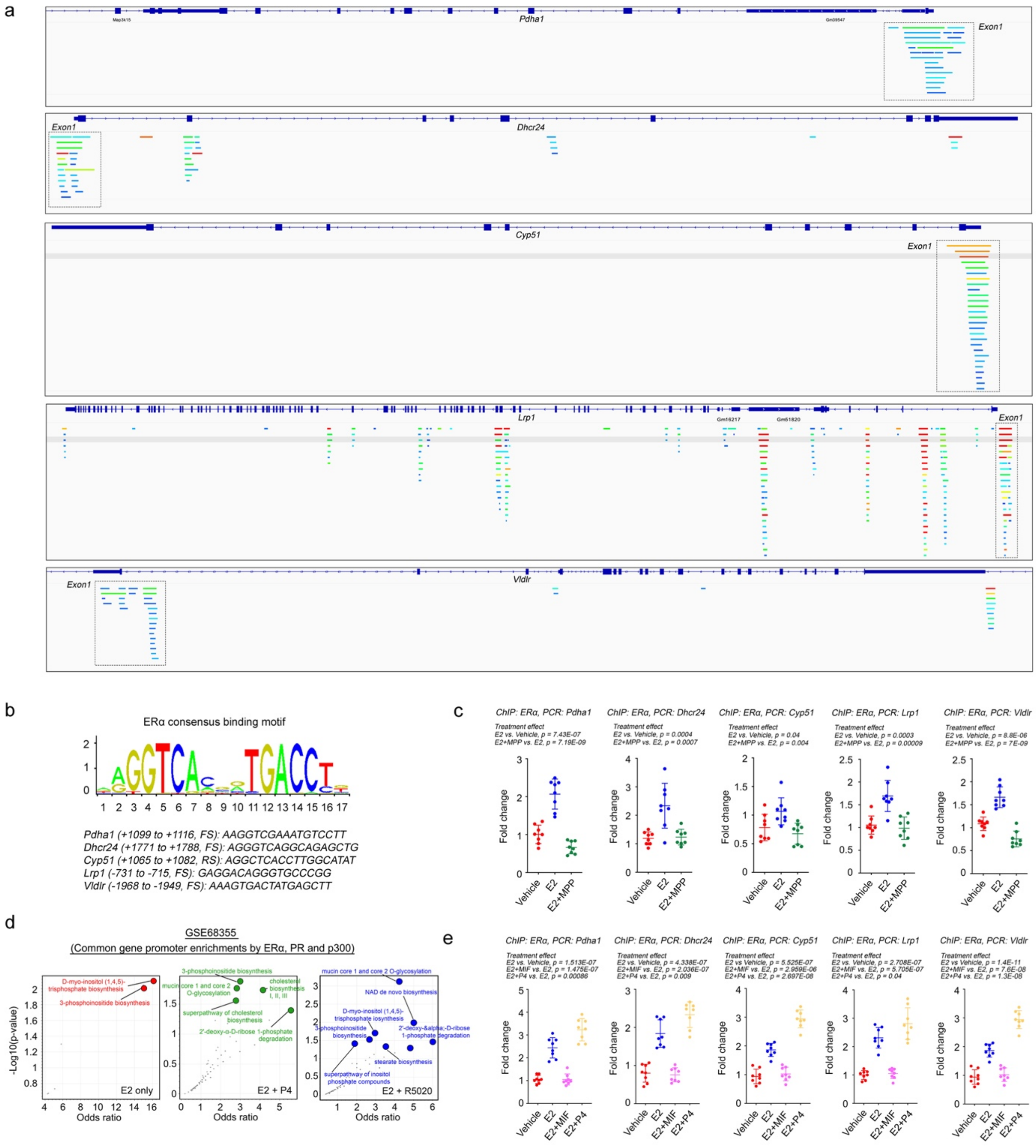

Supplementary Fig. 7. Additional data related to ChIP–PCR analysis. a, Analyses of mouse *Pdha1, Dhcr24, Cyp51, Lrp1* and *Vldlr* gene promoters for ERα binding to ChIP-Atlas. **b,** ERα consensus binding motifs identified on the *Pdha1, Dhcr24, Cyp51, Lrp1* and *Vldlr* genes. FS, forward strand of DNA; RS, reverse strand of DNA. **c,** Quantification of the band intensities from the ChIP–PCR data shown in Fig. 4f (n=8; one-way ANOVA with Tukey's multiple comparisons test). **d,** Pathway enrichment analysis of common gene promoters coregulated by ERα, PR and p300 under various treatment conditions. Data from GSE68355[5] were extracted and reanalyzed on Enrichr[1] (two-tailed Fisher's exact test with correction). **e,**

Quantification of the band intensities from the ChIP–PCR data shown in Figure 4i (n=8; one-way ANOVA with Tukey's multiple comparisons test). N represents biological replicates. The values represent the mean ± s.d. Source data are provided as a Source Data file.

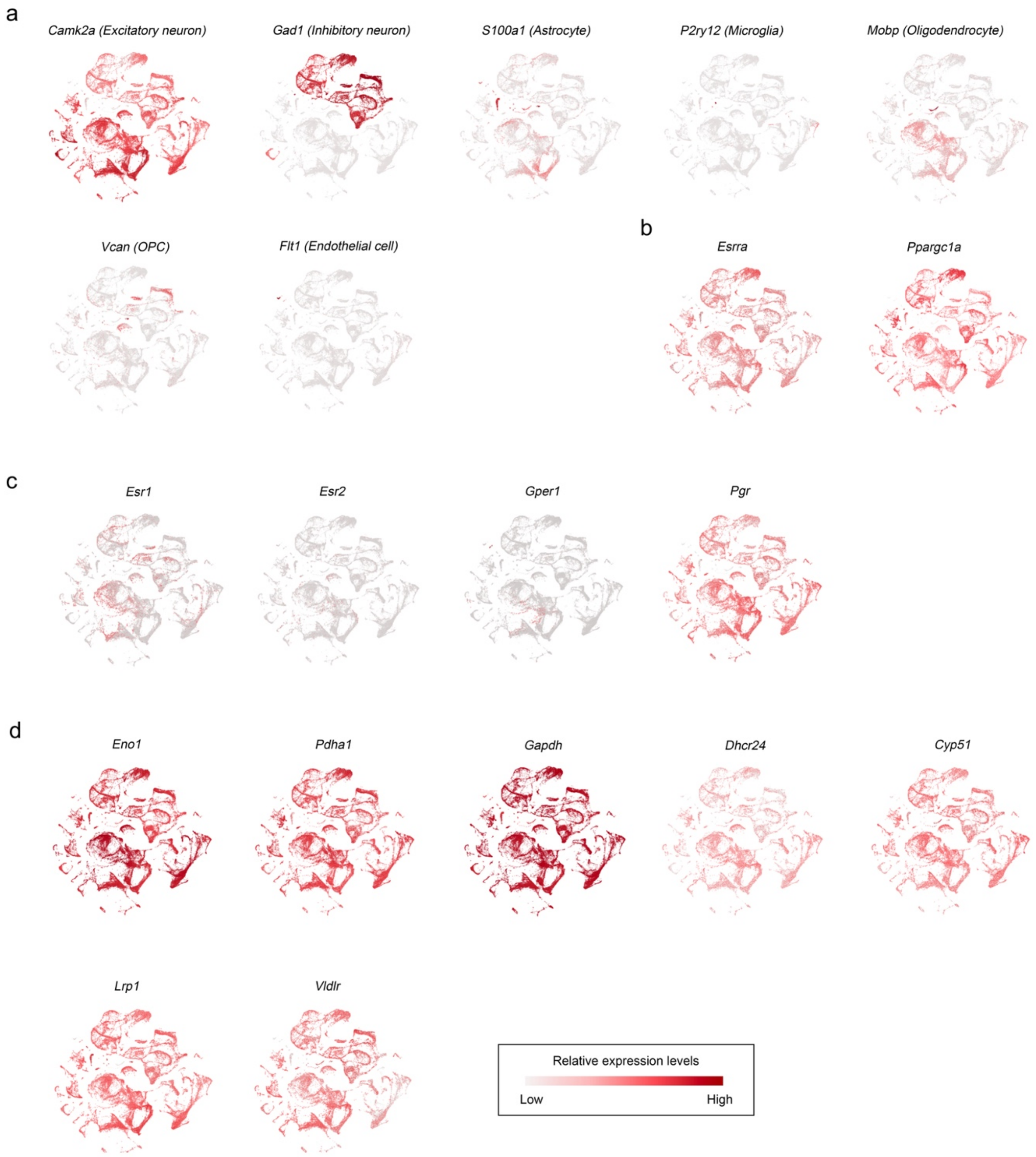

Supplementary Fig. 8. Scatter plots generated from the Transcriptomics Explorer of the Allen Brain Map reveal the relative expression levels of target genes of interest in various mouse brain cell types within the whole cortex and hippocampus regions.

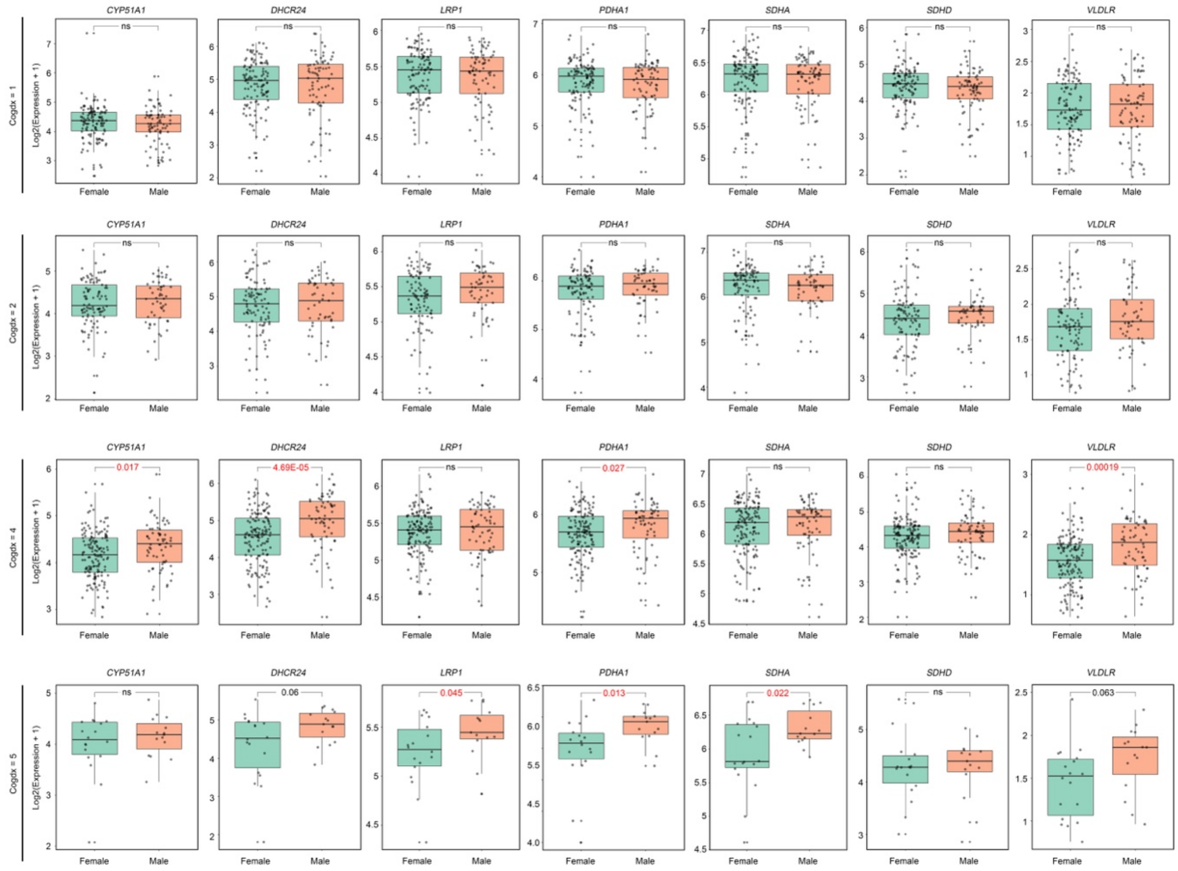

Supplementary Fig. 9. Tukey plots for comparisons of the expression levels of ERα-targeted metabolic genes in ROSMAP brain samples harvested from patients of different biological sexes with different cognitive scores (Cogdx) (ND: male n = 133 and female n = 219; LOAD: male n = 88 and female n = 173; two-tailed unpaired t test). N represents biological replicates. The values represent the mean ± s.d.

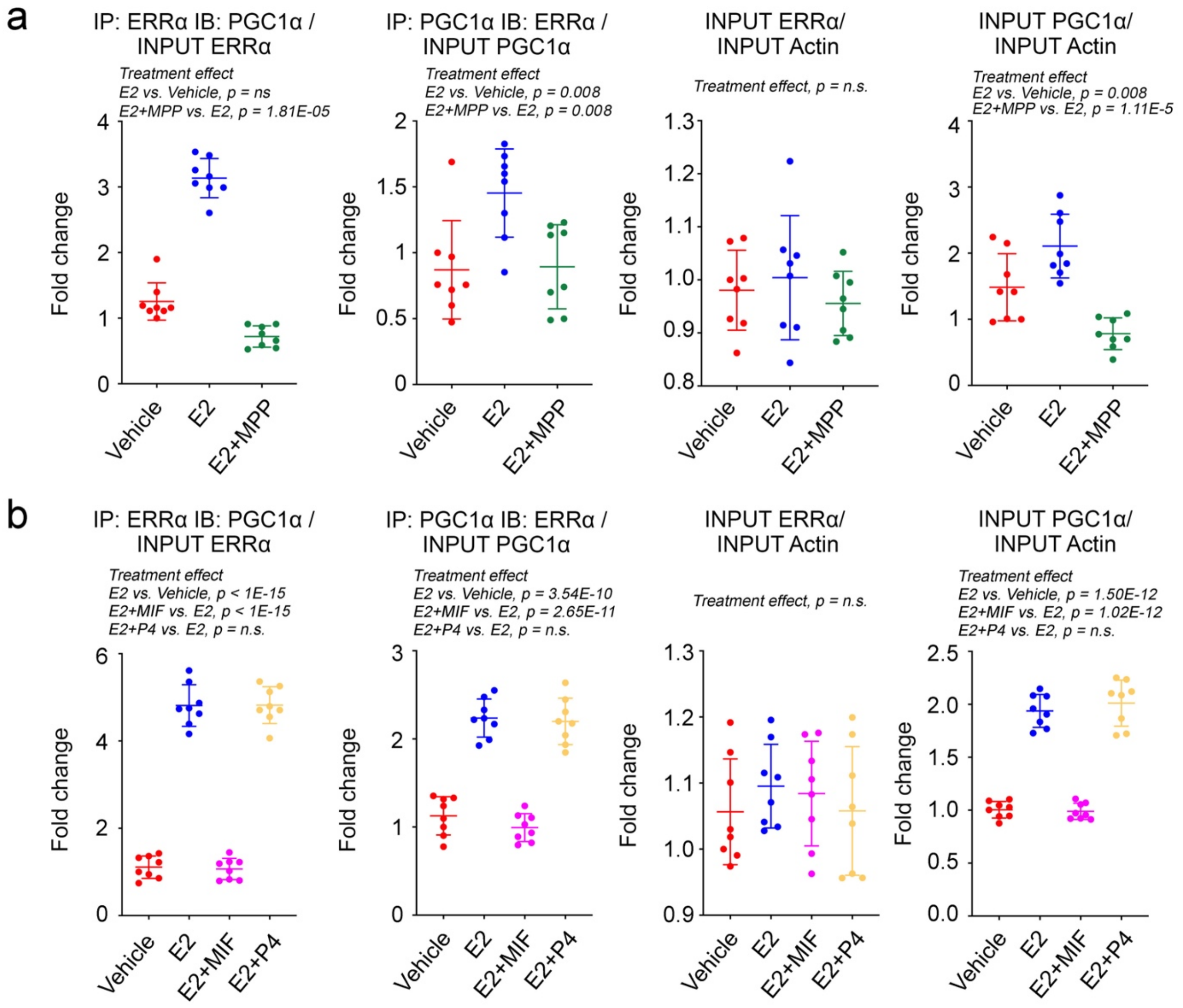

Supplementary Fig. 10. Quantification of the Western blot data, a. In the left panel of Figure 4l (n=8, one-way ANOVA with Tukey's multiple comparisons test or Holm–Šídák's multiple comparisons test for all except for the quantification of IP:ERRα IB:PGC1A/INPUT ERRα, for which a Kruskal–Wallis test with Dunn's multiple comparisons test was used). N represents biological replicates. **b.** Right panel of Figure 4l (n=8; one-way ANOVA with Šídák's multiple comparisons test). N denotes the biological replicates. The values represent the mean ± s.d. Source data are provided as a Source Data file.

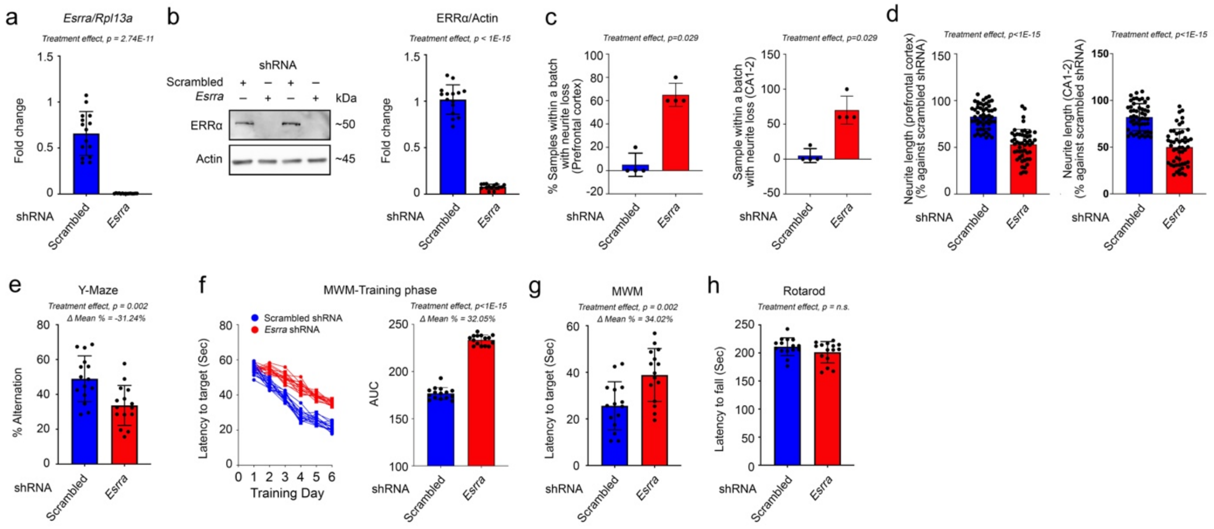

Supplementary Fig. 11. Additional data related to AAV-shRNA *in vivo* experiments. a, Gene expression levels of *Esrra* in targeted brain tissues harvested from the tested animals after the entire treatment paradigm (n=15; two-tailed unpaired t test). **b,** Representative Western blots reveal how treatment affects total ERRα protein levels in targeted brain tissue regions. The data are shown on the right (n=15; two-tailed unpaired t test). **c,** Quantification of the percentage of samples within a cohort with neurite loss in the prefrontal cortex and hippocampal regions (i.e., CA1 and CA2) was performed (n= 4 cohorts with a total of 20 biological replicates; Mann–Whitney test). **d,** Quantification of neurite length in different treatment groups normalized to that in the scrambled shRNA control group (n=10 biological replicates; 5–6 experimental repeats; two-tailed unpaired t test). **e-h,** Behavioural tests conducted on test animals after the treatments. The parameters assessed included **e,** the percentage of alternation in the Y-maze paradigm; **f-g,** the latency to target in the Morris water maze test during both the training phase and the probe trial; and **h,** the latency to fall in the rotarod test (n=15; two-tailed unpaired t test). N represents biological replicates. The values represent the mean ± s.d. Source data are provided as a Source Data file.

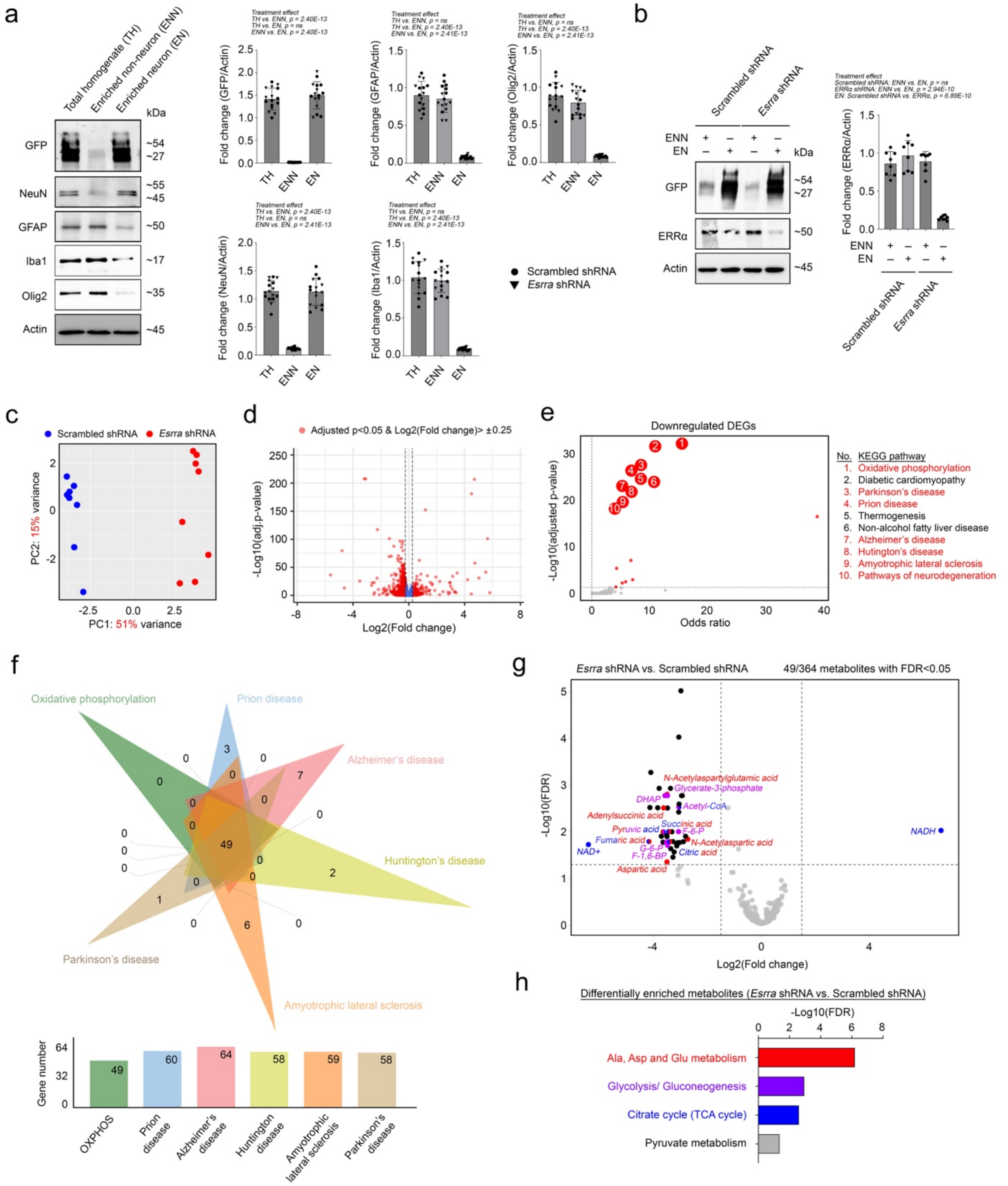

Supplementary Fig. 12. Molecular profiling of enriched neuronal populations from cerebral cortex tissues harvested from control or *Esrra*-KD C57BL/6 mice. a-b. Representative immunoblots showing successful enrichment of neuronal and nonneuronal glial cell populations from brain tissues of various samples (n=16; one-way ANOVA with Tukey's multiple comparison test). **b.** GFP signals were also highly enriched in the neuronal population, supporting the conclusion of **a.** (n=8; one-way ANOVA with

Tukey's multiple comparisons test). **c,** Principal component plot (PCA) indicating that enriched neuronal samples from the two groups were distinctly clustered (n=8). **d,** Volcano plot reflecting an overview of the DEGs resulting from comparisons between the two groups of enriched neurons [adjusted p-value <0.05; log2(fold change) > |0.25|] (two-tailed Wald test with Benjamini–Hochberg correction). **e,** Functional enrichment analysis of all downregulated DEGs found in **d.** on Enrichr[1] (two-tailed Fisher's exact test with correction). **f**, Venn diagram revealing the number of genes enriched in OXPHOS that are common to other neurodegenerative disease pathways. **g,** Volcano plot summarizing significantly altered metabolites in neuronal cells enriched from brain tissues of *Esrra*-KD versus control group animals (n=8; two-sided Welch multiple unpaired t test with Benjamini, Krieger, and Yekutieli correction). **h,** Differentially changed metabolites were clustered and analysed by metabolite set enrichment analysis conducted on MetaboAnalyst[3]. Unique colour codes of metabolites in **g.** are matched to the corresponding pathways (Globaltest with Bonferroni correction[4]). N represents biological replicates. The values represent the mean ± s.d. Source data are provided as a Source Data file.

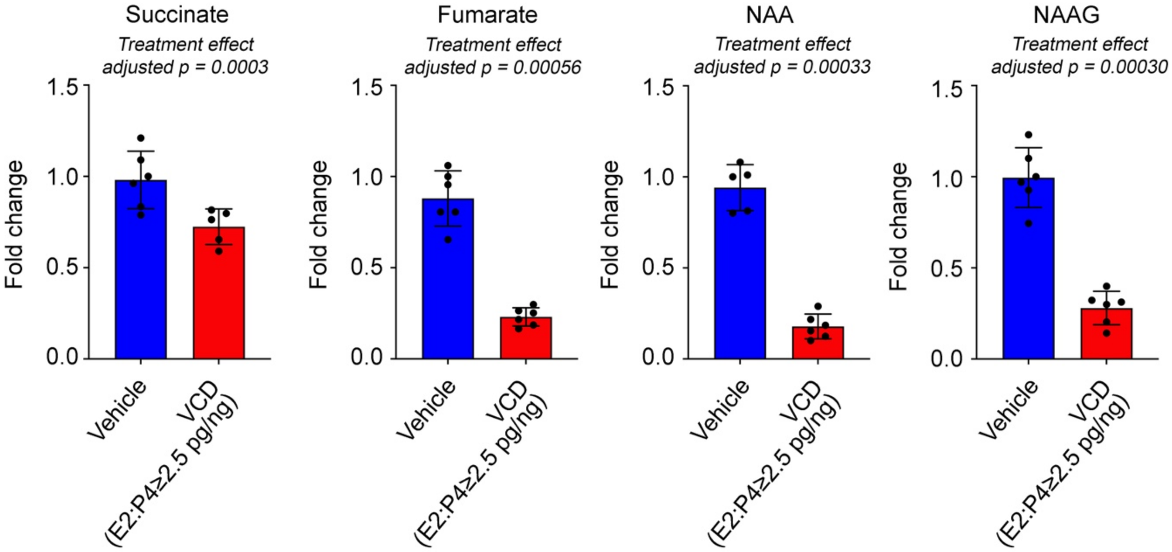

Supplementary Fig. 13. Levels of key metabolites in brain tissues harvested from VCD-treated animals. Data were extracted from the unbiased metabolomics study presented in Figure 4a (n=6; two-sided Welch multiple unpaired t test with Benjamini, Krieger, and Yekutieli correction). N represents biological replicates. The values represent the mean ± s.d. Source data are provided as a Source Data file.

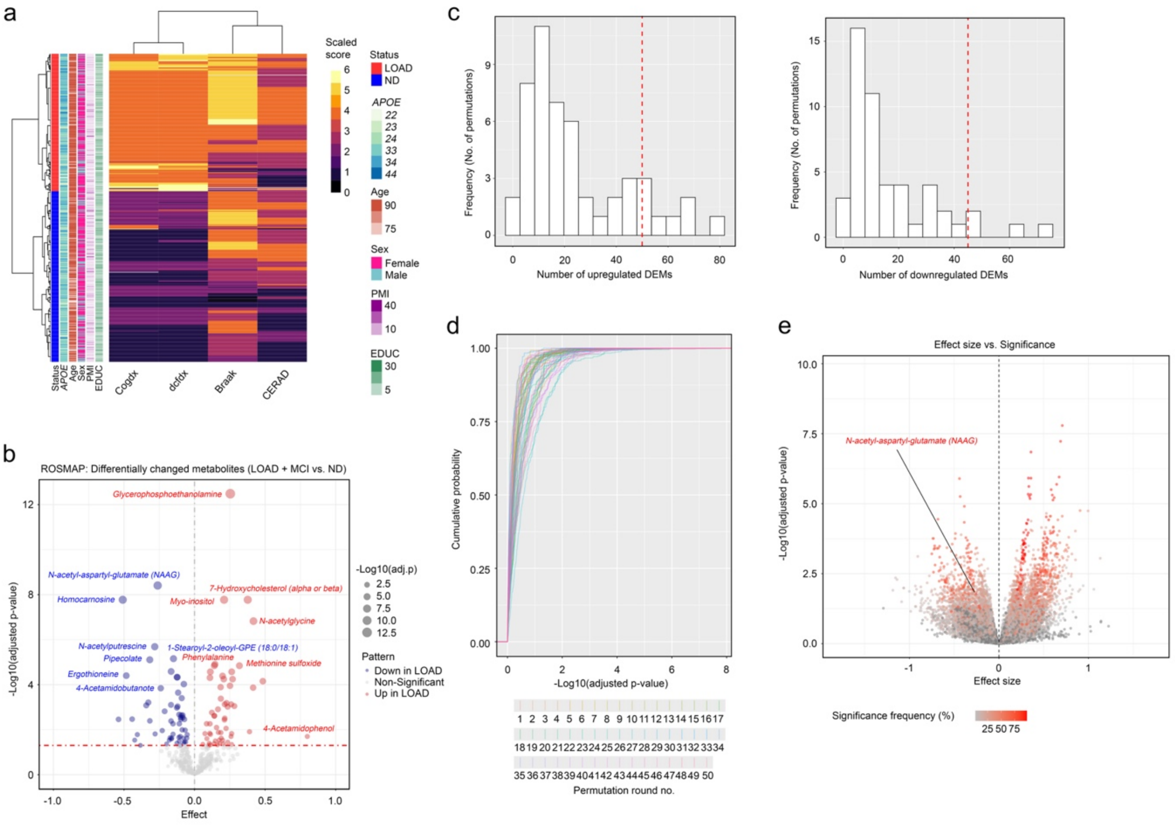

Supplementary Fig. 14. Permutation analysis confirmed that the differentially expressed metabolites found in female LOAD subjects in the ROSMAP cohort are not biased by the preexisting difference in sample numbers. **a,** The disease status of the ROSMAP study samples was defined on the basis of multiple clinicopathological parameters (x-axis). **b,** In the analysis performed between all LOAD and ND samples regardless of their biological sex, differential expression analysis was performed for a given metabolite by fitting a generalized linear model (GLM) to estimate the association between the expression of a metabolite and its disease status while adjusting for covariates using Gaussian linear regression. Multiple testing correction was then performed on the calculated p value using the Benjamini–Hochberg (BH) procedure, controlling the false discovery rate (FDR) to obtain the adjusted p value. **c,** Given that the number of female samples was significantly greater than that of male samples in both the LOAD (male: 62; female: 161) and ND groups (male: 86; female: 191), a permutation analysis was conducted. This test involved downsizing the sample numbers to match the count of male-specific samples. The experimental groups were formed by random sampling of data within the populations, and statistical inferences were applied to these randomized, downsized datasets. This process was repeated 50 times by randomly eliminating female samples to validate that any observed differences in differentially expressed metabolites (DEMs) were not due to preexisting sample count disparities. The distribution of upregulated and downregulated permuted DEMs from the permutation analysis was visualized using a histogram. The number of DEMs identified in the original female-specific comparison with all available samples is depicted using a red dotted line in the respective histograms. **d,** Although the original full analysis revealed more DEMs than the permutation analysis did, an empirical cumulative distribution function (ECDF) plot demonstrated

that the randomized datasets had similar distributions and cumulative probabilities at each value, despite variations in each permutation. **e,** Volcano plot illustrating the frequency of metabolite significance across permutations. Metabolites with higher recurrence frequencies in the 50 rounds of permutation analysis are highlighted in red.

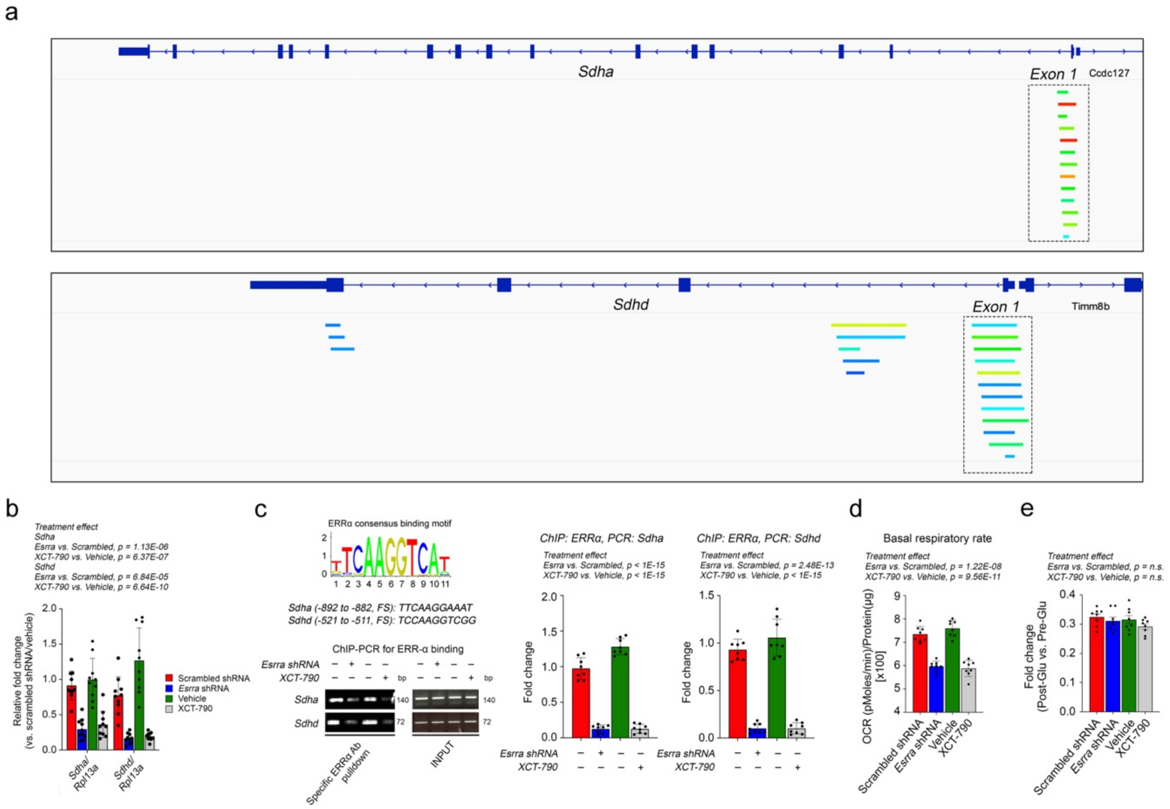

Supplementary Fig. 15. Analyses of the succinate dehydrogenase subunits *Sdha* and *Sdhd*. a, Analysis of the binding of the mouse *Sdha* and *Sdhd* gene promoters to ERRα by ChIP-Atlas. **b,** qPCR analysis of *Sdha* and *Sdhd* transcript levels in primary cortical neurons presilenced with *Esrra* shRNA (or scrambled shRNA control) or pretreated with 400 nM XCT-790 (or vehicle) for 120 hours (n=10; one-way ANOVA with Tukey's multiple comparison test). **c,** ERRα consensus binding motifs identified on the *Sdha* and *Sdhd* gene promoters. FS, forwards strand of DNA; RS, reverse strand of DNA. The results of the quantification of the band intensities from the ChIP–PCR data are shown on the right (n=8; one-way ANOVA with Tukey's multiple comparison test). **d,** Basal respiratory rates as calculated from the Mito Stress Test as presented in Fig. 6k (n=8; one-way ANOVA with Tukey's multiple comparisons test). **e,** The relative abundance of intracellular ATP measured immediately after the removal of glutamate (Signal ratio: Post-$E_{2,135s}$/Pre-$E_{1,993sec}$) as presented in Fig. 6l (n=8; one-way ANOVA with Tukey's multiple comparisons test). N represents biological replicates. The values represent the mean ± s.d. Source data are provided as a Source Data file.

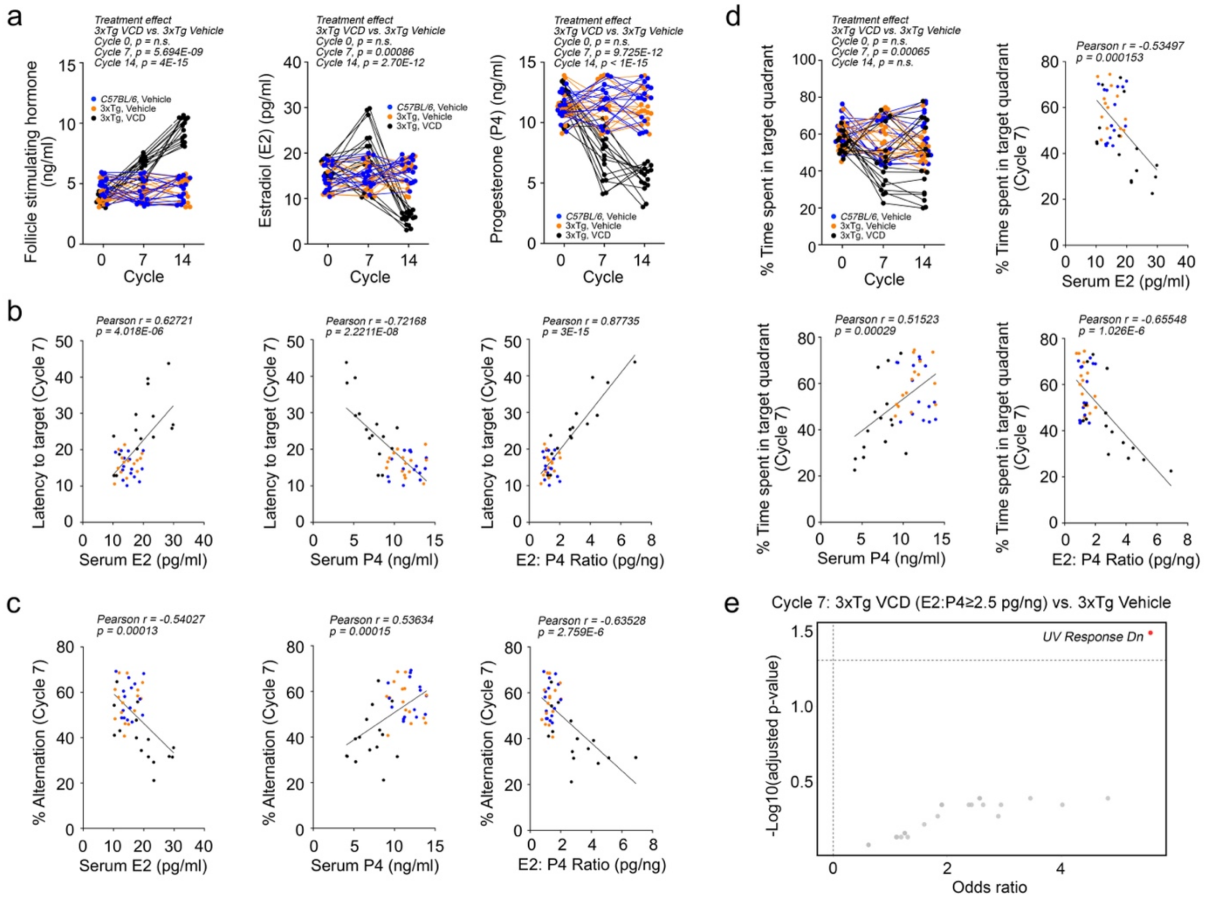

Supplementary Fig. 16. Additional data related to behavioural tests and their correlations with plasma hormonal changes in 3xTg animals. a, Changes in plasma levels of follicle-stimulating hormone (n=15, two-way ANOVA with Tukey's multiple comparisons test), oestradiol (E2) (n=15, n=15, two-way ANOVA with Šídák's multiple comparisons test), and progesterone (P4) (n=15, two-way ANOVA with Šídák's multiple comparisons test) during the proestrus phase of cycles 0, 7 and 14. **b-d,** Comparison of the strength of correlations between performance from various behavioural paradigms, including **b,** latency to target of the MWM paradigm (n=15, Pearson correlation test; **c,** percentage alternation of the Y-maze paradigm (n=15, Pearson correlation test; and **d,** time spent in the target quadrant of the MWM paradigm against either plasma E2, plasma P4 or the E2:P4 ratio (n=15, two-way ANOVA with Tukey's multiple comparisons test, Pearson correlation test). **e,** Pathway enrichment analysis of upregulated DEGs in brain tissues harvested from VCD-treated (E2:P4 ≥2.5 pg/ng) and vehicle-treated 3xTg mice at cycle 7 on Enrichr[1] (two-tailed Fisher's exact test with correction). N represents biological replicates. The values represent the mean ± s.d. Source data are provided as a Source Data file.

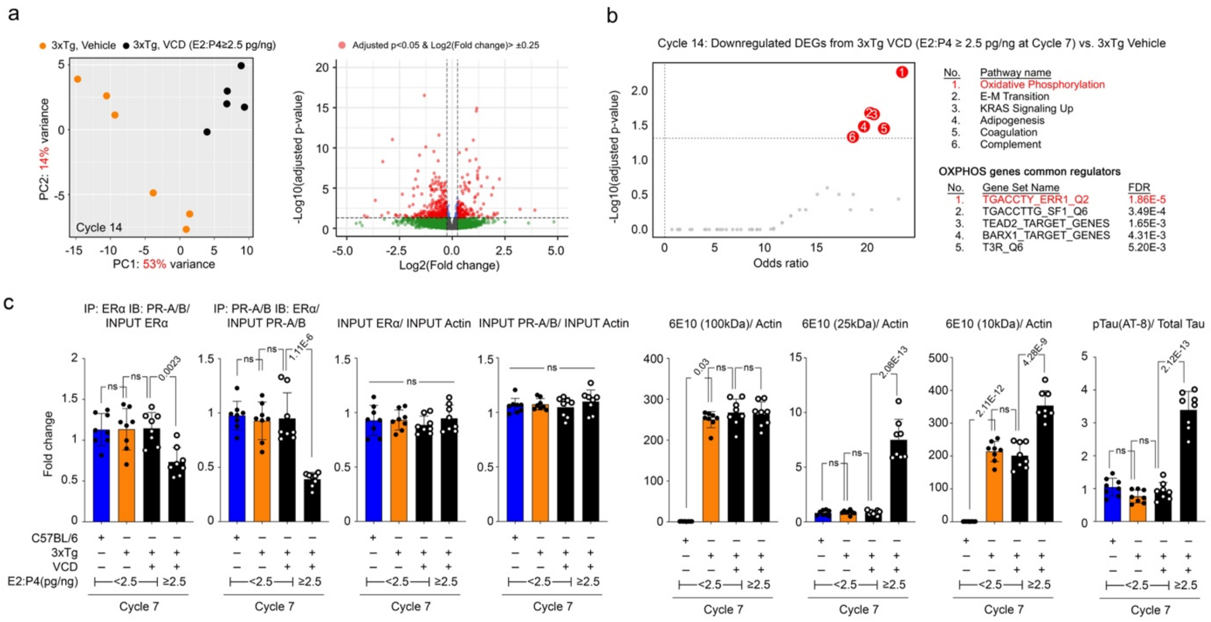

Supplementary Fig.17. Bulk transcriptomic analysis of brain tissues harvested from VCD-treated 3xTg mice at Cycles 14–15 and immunoblotting experiment quantification. a, Left: principal component plot (PCA) showing the distinct clustering of 3xTg brain samples from the vehicle (n=6) and VCD-treated groups (i.e., E2:P4≥2.5 pg/ng) (n=5) harvested at Cycles 14–15. Right: Volcano plot indicating that a total of 767 up- and 1305 downregulated transcripts were significantly different between the two groups (adjusted p-value <0.05; log2(fold change) > |0.25|) (two-sided Wald test with Benjamini–Hochberg correction). **b,** Pathway enrichment analysis of downregulated DEGs in brain tissues harvested from VCD-treated (E2:P4 ≥2.5 pg/ng) and vehicle-treated 3xTg mice at Cycles 14–15 conducted on Enrichr[1] (two-sided Fisher's exact test with correction). Top 5 common transcription regulators of OXPHOS enriched in the oxidative phosphorylation (OXPHOS) pathway predicted on the GSEA platform[6] with reference to the TFT_LEGACY subset of TFT (Mann–Whitney rank sum test with corrections)[7]. **c,** Quantification of the immunoblotting results presented in Figure 7i (n=8; one-way ANOVA with Tukey's multiple comparisons test, except that for 6E10 (100 kDa)/Actin, the Kruskal–Wallis test with Dunn's multiple comparisons test was used). N represents biological replicates. The values represent the mean ± s.d. Source data are provided as a Source Data file.

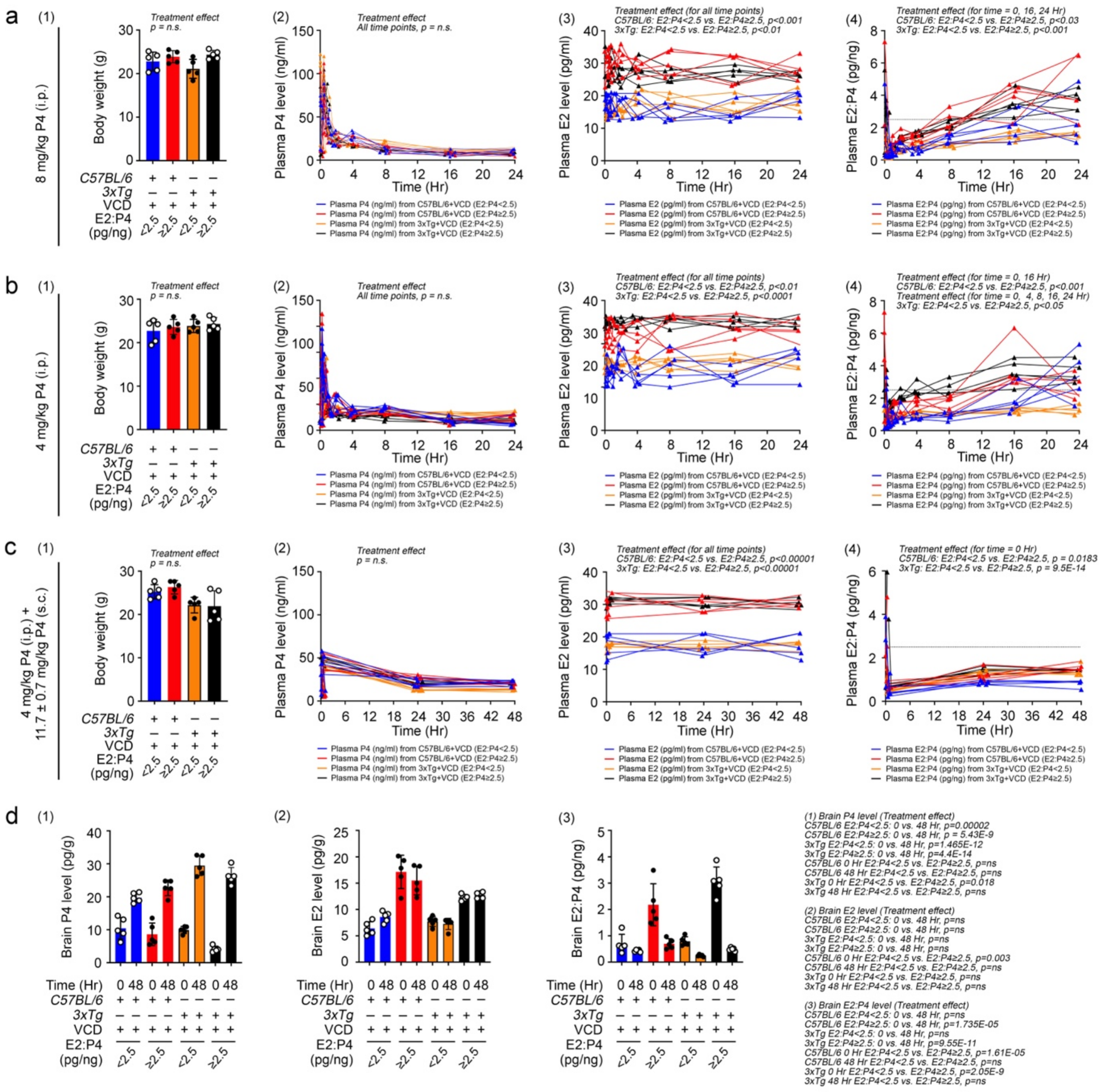

Supplementary Fig. 18. Changes in plasma and brain E2 and P4 levels upon P4 administration in C57BL/6 and 3xTg animals. a, (1) Body weights of the experimental animals (n=5; one-way ANOVA with Tukey's multiple comparisons test), (2) plasma P4 concentration (n=5; two-way ANOVA with Šídák's multiple comparisons test), (3) plasma E2 concentration (n=5; two-way ANOVA with Šídák's multiple comparisons test) and (4) E2:P4 ratio (n=5; two-way ANOVA with Šídák's multiple comparisons test) in response to a single intraperitoneal dose of 8 mg/kg P4. **b,** (1) Body weights of the experimental animals (n=5, one-way ANOVA with Tukey's multiple comparisons test), (2) plasma P4 concentration (n=5, two-way ANOVA with Šídák's multiple comparisons test), (3) plasma E2 concentration (n=5, two-way ANOVA with Šídák's multiple comparisons test) and (4) E2:P4 ratio (n=5, two-way ANOVA with Šídák's multiple comparisons test) in response to a single intraperitoneal dose of 4 mg/kg P4. **c,** (1) Body weights of the experimental animals (n=5, one-way ANOVA with Tukey's multiple comparisons test), (2) plasma P4 concentration (n=5, two-way ANOVA with

Šídák's multiple comparisons test), (3) plasma E2 concentration (n=5, two-way ANOVA with Šídák's multiple comparisons test) and (4) E2:P4 ratio (n=5, two-way ANOVA with Šídák's multiple comparisons test) in response to a single intraperitoneal dose of 4 mg/kg P4 as well as subcutaneous administration of 11.7 ± 0.7 mg/kg P4. **d,** (1) Brain levels of P4 (n=5, one-way ANOVA with Šídák's multiple comparisons test), (2) E2 levels (n=5, Kruskal–Wallis test with Dunn's multiple comparisons test) and (3) E2:P4 ratios (n=5, one-way ANOVA with Šídák's multiple comparisons test) in response to a single intraperitoneal dose of 4 mg/kg P4 as well as subcutaneous administration of 11.7 ± 0.7 mg/kg P4. N represents biological replicates. The values represent the mean ± s.d. Source data are provided as a Source Data file.

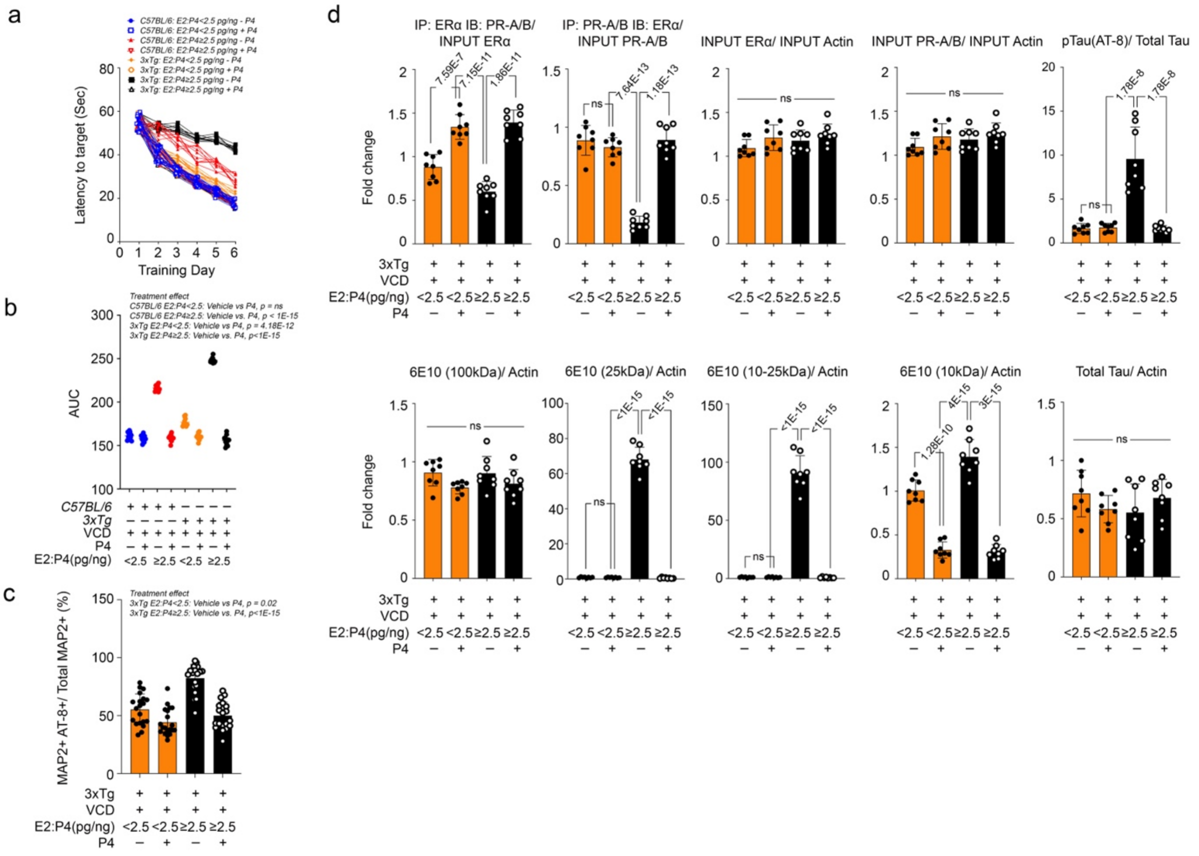

Supplementary Fig. 19. Additional data related to behavioural tests, immunohistochemistry analysis, and immunoblotting experiments in test animals subjected to 60 days of P4 supplementation at Cycles 14–15. a-b, a. Latencies to target curves during the 6-day training in the MWM paradigm conducted at time after P4 administration. **b.** The areas under the curve were calculated (n=10; one-way ANOVA with Holm–Šídák's multiple comparisons test). **c,** Quantification of the percentage of AT-8-positive neurons in prefrontal cortex tissues harvested from test animals (n=5; 4 experimental repeats; one-way ANOVA with Tukey's multiple comparison test). **d,** Quantification of the immunoblotting results presented in Figure 7n (n=8; one-way ANOVA with Holm–Šídák's multiple comparisons test for all except total tau/actin, for which one-way ANOVA with Tukey's multiple comparisons test was used). N represents biological replicates. The values represent the mean ± s.d. Source data are provided as a Source Data file.

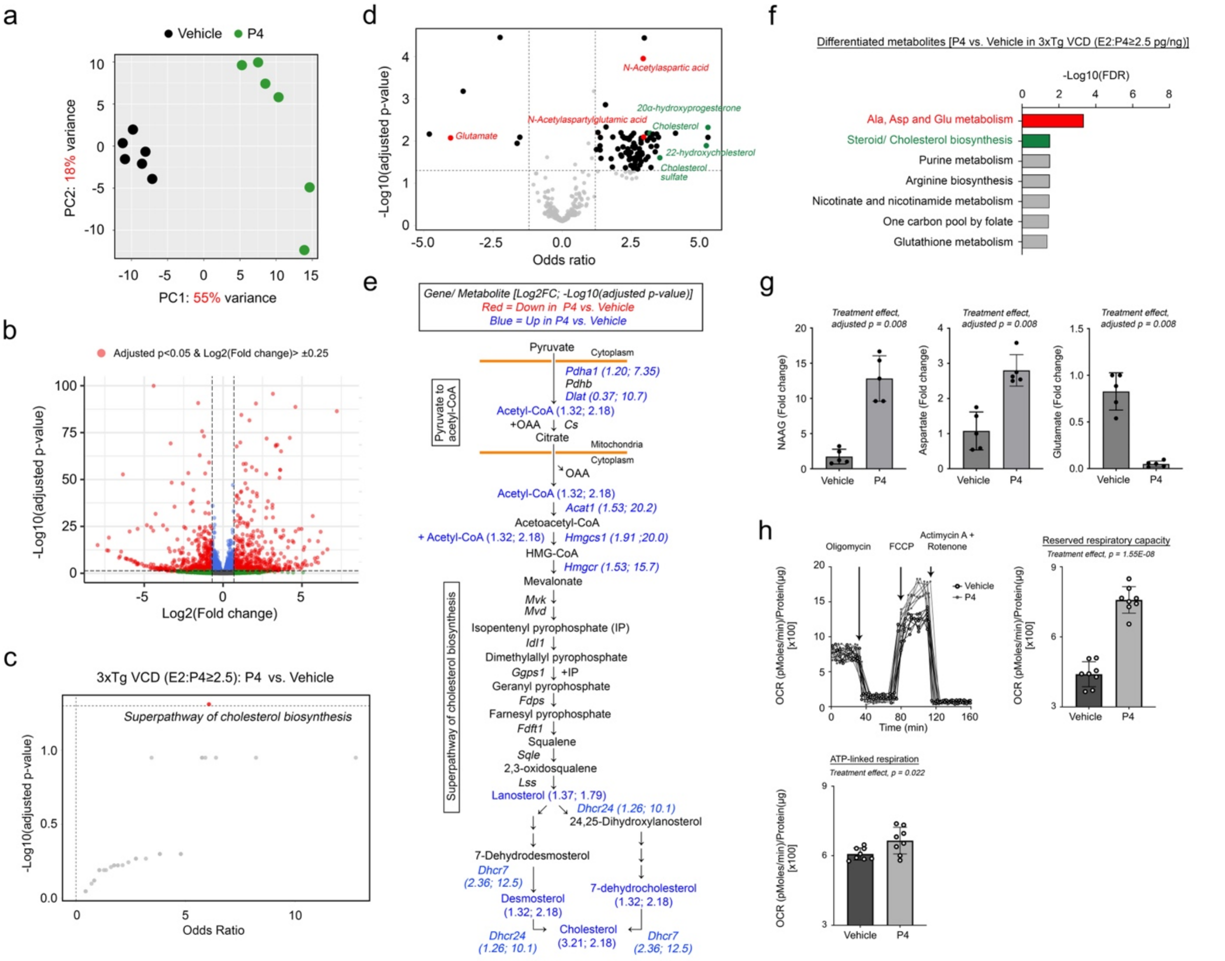

Supplementary Fig. 20. Bulk transcriptomic and metabolomic analyses of brain tissues harvested from VCD-treated 3xTg mice subjected to 60 days of P4 supplementation at cycles 14–15. a, Principal component plot (PCA) indicating that brain samples harvested from VCD (E2:P4 ≥ 2.5 pg/ng) ± P4-treated 3xTg mice at cycles 14–15 were distinctly clustered (N=5–6). **b,** Volcano plot indicating that a total of 994 up- and 1010 downregulated transcripts were significantly different between the two groups (adjusted p-value <0.05; log2(fold change) > |0.25|] (two-sided Wald test with Benjamini–Hochberg correction). **c,** Pathway enrichment analysis of upregulated DEGs identified on Enrichr[1] (two-sided Fisher's exact test with correction). **d,** Volcano plot summarizing significantly altered metabolites in neuronal cells enriched from brain tissues harvested from VCD (E2:P4≥2.5 pg/ng) ± P4-treated 3xTg mice at cycles 14–15 (n=5; two-sided Welch multiple unpaired t test with Benjamini, Krieger, and Yekutieli correction). **e,** Diagram illustrating the fate of glucose carbon in the biosynthesis of cholesterol. Gene expression of enzymes identified in **a-b.** and metabolites upregulated in the P4-treated group, as identified in **d,** are shown in blue; downregulated enzymes and metabolites are shown in red. Those with no significant change are in black. **f,** Differentially changed metabolites identified in **d.** were clustered and analysed by metabolite set enrichment analysis on MetaboAnalyst[3]. Unique colour codes of the metabolites in **d.** are matched to the corresponding pathways (global test with Bonferroni correction[4]). **g,** Targeted analyses of the levels of specific metabolites, including NAAG, aspartate, and glutamate, in response to P4 administration (n=5; two-sided Welch's multiple unpaired t test

with Benjamini, Krieger, and Yekutieli correction). **h,** Raw profiles of mito stress assays in harvested brain tissues. Reserved respiratory capacity and ATP-linked respiration were calculated (n=8; two-tailed unpaired t test). N represents biological replicates. The values represent the mean ± s.d. Source data are provided as a Source Data file.

References

1 Chen, E. Y. *et al.* Enrichr: interactive and collaborative HTML5 gene list enrichment analysis tool. *BMC Bioinformatics* **14**, 128 (2013). https://doi.org:10.1186/1471-2105-14-128

2 Mathys, H. *et al.* Single-cell transcriptomic analysis of Alzheimer's disease. *Nature* **570**, 332-337 (2019). https://doi.org:10.1038/s41586-019-1195-2

3 National Academies of Sciences, E., and Medicine; Health and Medicine Division; Behavioral and Social Sciences and Education; Board on Health Sciences Policy; Board on Behavioral, Cognitive, and Sensory Sciences; Committee on Research Priorities for Preventing and Treating Alzheimer's Disease and Related Dementias. in *Preventing and Treating Dementia: Research Priorities to Accelerate Progress The National Academies Collection: Reports funded by National Institutes of Health* (eds O. C. Yost, A. Downey, & T. Powell) (2025).

4 Xia, J. & Wishart, D. S. MSEA: a web-based tool to identify biologically meaningful patterns in quantitative metabolomic data. *Nucleic Acids Res* **38**, W71-77 (2010). https://doi.org:10.1093/nar/gkq329

5 Mohammed, H. *et al.* Progesterone receptor modulates ERalpha action in breast cancer. *Nature* **523**, 313-317 (2015). https://doi.org:10.1038/nature14583

6 Subramanian, A. *et al.* Gene set enrichment analysis: a knowledge-based approach for interpreting genome-wide expression profiles. *Proc Natl Acad Sci U S A* **102**, 15545-15550 (2005). https://doi.org:10.1073/pnas.0506580102

7 Xie, X. *et al.* Systematic discovery of regulatory motifs in human promoters and 3' UTRs by comparison of several mammals. *Nature* **434**, 338-345 (2005). https://doi.org:10.1038/nature03441
