# Supplementary material for: Perimenopausal state oestradiol to progesterone imbalance drives Alzheimer’s risk via ERRα dysregulation and energy dyshomeostasis
Source: Nat Commun. 2025 Nov 22;16:11546. doi: 10.1038/s41467-025-66726-4 (PMC12748969; doi:10.1038/s41467-025-66726-4)
Supplement: Supplementary file 2 — Description of Additional Supplementary Files [file 41467_2025_66726_MOESM2_ESM.pdf]

Description of Additional Supplementary Files

File Name: Supplementary Data 1
Description: Sample information from the ROSMAP metadata analyses and immunohistology analysis.

File Name: Supplementary Data 2
Description: Special reagents, antibodies and primer lists.
