# Supplementary material for: Perimenopausal state oestradiol to progesterone imbalance drives Alzheimer’s risk via ERRα dysregulation and energy dyshomeostasis
Source: Nat Commun. 2025 Nov 22;16:11546. doi: 10.1038/s41467-025-66726-4 (PMC12748969; doi:10.1038/s41467-025-66726-4)
Supplement: Supplementary file 5 — Reporting Summary [file 41467_2025_66726_MOESM5_ESM.pdf]

nature portfolio

Corresponding author(s): Kim Hei-Man CHOW

Last updated by author(s): 2025/10/23

Reporting Summary

Nature Portfolio wishes to improve the reproducibility of the work that we publish. This form provides structure for consistency and transparency in reporting. For further information on Nature Portfolio policies, see our Editorial Policies and the Editorial Policy Checklist.

Statistics

For all statistical analyses, confirm that the following items are present in the figure legend, table legend, main text, or Methods section.

n/a	Confirmed	
☐	☒	The exact sample size (*n*) for each experimental group/condition, given as a discrete number and unit of measurement
☐	☒	A statement on whether measurements were taken from distinct samples or whether the same sample was measured repeatedly
☐	☒	The statistical test(s) used AND whether they are one- or two-sided<br>*Only common tests should be described solely by name; describe more complex techniques in the Methods section.*
☒	☐	A description of all covariates tested
☐	☒	A description of any assumptions or corrections, such as tests of normality and adjustment for multiple comparisons
☐	☒	A full description of the statistical parameters including central tendency (e.g. means) or other basic estimates (e.g. regression coefficient) AND variation (e.g. standard deviation) or associated estimates of uncertainty (e.g. confidence intervals)
☐	☒	For null hypothesis testing, the test statistic (e.g. *F*, *t*, *r*) with confidence intervals, effect sizes, degrees of freedom and *P* value noted<br>*Give P values as exact values whenever suitable.*
☒	☐	For Bayesian analysis, information on the choice of priors and Markov chain Monte Carlo settings
☒	☐	For hierarchical and complex designs, identification of the appropriate level for tests and full reporting of outcomes
☐	☒	Estimates of effect sizes (e.g. Cohen's *d*, Pearson's *r*), indicating how they were calculated

Our web collection on statistics for biologists contains articles on many of the points above.

Software and code

Policy information about availability of computer code

Data collection	The following commercial software was used to collect data:<br>Magellan (TECAN plate reader); LAS X (Leica); Seahorse Wave Desktop software (Agilent); LightCycler 480 Software (Roche); GenePix Pro6.0 (Molecular Devise); Agilent MassHunter software (Agilent); Freeze Frame Software; Freeze Frame software (Actimetrics); Smart 3.0 video tracking system (Panlab, Harvard Apparatus); AB Sciex Analyst 1.4.2 (Applied Biosystems); HiSeq X Ten platform (Illumina), NIS-Elements software (Nikon), LAS X Life Science Microscope software (Leica); PClamp analysis software, Clampex 10.7 and Clampfit (Molecular Devices), Minianalysis software (Synaptosoft).
Data analysis	The following commercial software was used to analyze data:<br>Microsoft Excel; Morpheus platform (Broad Institute); BBrowser2.7.48 (BioTuring Inc); (GEO2R (NIH); Prism 10 (Graphpad); Seahorse Wave Desktop software (Agilent); Metabolite Set Enrichment Analysis (Metaboanalyst); Gene Set Enrichment Analysis (Broad Institute); Agilent OpenLab CDS (ChemStation Edition); Freeze Frame software (Actimetrics); Smart 3.0 video tracking system (Panlab, Harvard Apparatus); Image J v1.8.0 (NIH); AB Sciex Analyst 1.4.2 (Applied Biosystems); LAS X Life Science Microscope software (Leica), MASCOT 2.3 software (Matrix Science), SWISS-MODEL algorithm (Swiss Institute of Bioinformatics), PyMOL software (DeLano Scientific), HADDOCK 2.4 (BioExcel Center of Excellence for Biomolecular Research), ChIP-Atlas (Kyoto University), Integrative Genomics Viewer (Broad Institute), JASPAR 2022 database (Elixir Norway, University of Copenhagen, Centre for Molecular Medicine and Therapeutics, London Institute of Medical Sciences and Centre for Molecular Medicine Norway), R studio (Posit Software), Enrichr (Icahn School of Medicine at Mount Sinai), FastQC V. 0.11.9 (Babraham Bioinformatics), STAR software (MIT), PClamp analysis software, Clampex 10.7 and Clampfit (Molecular Devices), Minianalysis software (Synaptosoft), AlphaFold2 on the ColabFold v1.4.0 (DeepMind).

For manuscripts utilizing custom algorithms or software that are central to the research but not yet described in published literature, software must be made available to editors and reviewers. We strongly encourage code deposition in a community repository (e.g. GitHub). See the Nature Portfolio guidelines for submitting code & software for further information.

Data

Policy information about availability of data

All manuscripts must include a data availability statement. This statement should provide the following information, where applicable:

- Accession codes, unique identifiers, or web links for publicly available datasets
- A description of any restrictions on data availability
- For clinical datasets or third party data, please ensure that the statement adheres to our policy

he bulk RNA-seq data generated in this study have been deposited in GEO Omnibus under the accession code GSE279885 (https://www.ncbi.nlm.nih.gov/geo/query/acc.cgi?acc=GSE279885.) The metabolomics data generated in this study have been included in Source Data file. Datasets utilized in this study were accessed with permission from the Accelerating Medicines Partnership® Program for Alzheimer's Disease (AMP® AD) platform under a signed agreement. The datasets were all generated from the ROSMAP cohort, these included the single cell transcriptomic data from the prefrontal cortex region (snRNAseqPFC_BA10) (Syn18485175)52 (https://www.synapse.org/Synapse:syn18485175); bulk RNA-seq data of dorsolateral prefrontal cortex (DLPFC), posterior cingulate gyrus (PCG), and anterior cingulate (AC) regions (syn3388564)131 (https://synapse.org/Synapse:syn3388564); and the non-targeted metabolomics data form DLPFC region (syn3157322) (https://www.synapse.org/Synapse:syn3157322). Key clinical and pathological features of de-identified subjects included these studies would be found in Supplementary Data 1. The definitive disease status (i.e., LOAD vs ND) of samples in the snRNA-seq dataset was provided by the original published paper and was adopted in this study52. For the bulk transcriptomics and metabolomics samples, definitive disease status was defined based on the Ward D2 hierarchical clustering method with reference to the CERAD score (i.e., a semi-quantitative measure of neuritic plaques); Braak staging score (i.e., semiquantitative measure of neurofibrillary tangles); Cogdx score (i.e., a clinical consensus diagnosis of cognitive status at time of death) and the Dcfdx score (i.e., a clinical diagnosis of the cognitive status). The ChIP-seq dataset of ER, PR and p300 in MCF7 breast cancer cell line treated with estrogen with or without progestins was extracted from GSE68355 (inside the Super Series GSE68359)13 (https://www.ncbi.nlm.nih.gov/geo/query/acc.cgi?acc=GSE68359). The protein structures used for docking analyses in this study can be found in the RCSB Protein Data Bank, including: 1XB7 for the X-ray structure of ERRα LBD in complex with a PGC1α peptide (https://doi.org/10.2210/pdb1XB7/pdb); 7E2E for the crystal structure of the ERRα LBD in complex with an agonist DS45500853 and a PGC-1α peptide (https://doi.org/10.2210/pdb7E2E/pdb) and 2PJL for the crystal structure of human ERRα in complex with a synthetic inverse agonist (https://doi.org/10.2210/pdb2PJL/pdb). The processed "Minimum dataset" data that are necessary to interpret, verify and extend the research in the article, are in "Source data files". Source data are provided with this paper. All analyses were carried out using freely available software packages.

Research involving human participants, their data, or biological material

Policy information about studies with human participants or human data. See also policy information about sex, gender (identity/presentation), and sexual orientation and race, ethnicity and racism.

Reporting on sex and gender	Not applicable.
Reporting on race, ethnicity, or other socially relevant groupings	Not applicable.
Population characteristics	Not applicable.
Recruitment	Not applicable.
Ethics oversight	Not applicable.

Note that full information on the approval of the study protocol must also be provided in the manuscript.

Field-specific reporting

Please select the one below that is the best fit for your research. If you are not sure, read the appropriate sections before making your selection.

☒ Life sciences ☐ Behavioural & social sciences ☐ Ecological, evolutionary & environmental sciences

For a reference copy of the document with all sections, see nature.com/documents/nr-reporting-summary-flat.pdf

Life sciences study design

All studies must disclose on these points even when the disclosure is negative.

Sample size	Sample size was calculated based on previous data (e.g. Chow et al., Nature Neuroscience, 2019 and other recent publications) assuming a power of 80 & and an alpha value of 0.05.
Data exclusions	No data exclusion was performed.
Replication	Number of replications are noted in the figure legends. Replicate data reproduced the initial observations.
Randomization	Litter mate mice were randomly assigned to control or experimental groups.
Blinding	Behavioral experiments were performed blinded and analyzed by different scientists. Blinding was not possible in each set of cell-based experiment as the same scientist performed the treatment and analyzes, however we had the replicating rounds of the same experiments performed by different scientists to confirm reproducibility.

Reporting for specific materials, systems and methods

We require information from authors about some types of materials, experimental systems and methods used in many studies. Here, indicate whether each material, system or method listed is relevant to your study. If you are not sure if a list item applies to your research, read the appropriate section before selecting a response.

Materials & experimental systems

n/a	Involved in the study	
☐	☒	Antibodies
☒	☐	Eukaryotic cell lines
☒	☐	Palaeontology and archaeology
☐	☒	Animals and other organisms
☒	☐	Clinical data
☒	☐	Dual use research of concern
☒	☐	Plants

Methods

n/a	Involved in the study	
☒	☐	ChIP-seq
☒	☐	Flow cytometry
☒	☐	MRI-based neuroimaging

Antibodies

Antibodies used	The complete list of antibodies used in this study can be found in the Supplementary Data 2.
Validation	All antibodies are listed in the Supplementary Data 2. These antibodies are also used in multiple previously published studies from our lab and others.

Animals and other research organisms

Policy information about studies involving animals; ARRIVE guidelines recommended for reporting animal research, and Sex and Gender in Research

Laboratory animals	C57BL/6J mice and 3xTg-AD were obtained from the Jackson Laboratory. Mouse colonies were maintained and bred in the Laboratory Animal Service Centre (LASEC) of CUHK, and their care was in accord with the institutional and Hong Kong guidelines
Wild animals	No wild animals were used in the present study.
Reporting on sex	This study focuses on the effects of menopausal transition in females, hence all test animals were therefore biologically female. For human data, both male and female samples were used in the metabolomics and bulk ROSMAP data analysis. Wherever applicable, the sex of the samples are indicated in the source data file, and all statistical analyses performed can be found in the source data files.
Field-collected samples	No field collected samples were used in the present study.
Ethics oversight	Mouse colonies were maintained and bred in the Laboratory Animal Services Centre of The Chinese University of Hong Kong (CUHK). All animal experimental protocols were approved by the Animal Ethics Committees at CUHK; and their care was in accord with the institutional and Hong Kong guidelines.

Note that full information on the approval of the study protocol must also be provided in the manuscript.

Plants

Seed stocks	Not applicable.
Novel plant genotypes	Not applicable.
Authentication	Not applicable.
